# Nutritional profile of ultra-processed foods consumed by children in Rio de Janeiro

**DOI:** 10.11606/s1518-8787.2020054001752

**Published:** 2020-08-28

**Authors:** Carine de Oliveira Avelar Anastácio, Juliana Martins Oliveira, Milena Miranda de Moraes, Jorginete de Jesus Damião, Inês Rugani Ribeiro de Castro

**Affiliations:** I Universidade do Estado do Rio de Janeiro Instituto de Nutrição Programa de Pós-Graduação em Alimentação, Nutrição e Saúde Rio de JaneiroRJ Brasil Universidade do Estado do Rio de Janeiro. Instituto de Nutrição. Programa de Pós-Graduação em Alimentação, Nutrição e Saúde. Rio de Janeiro, RJ, Brasil; II Universidade do Estado do Rio de Janeiro Instituto de Nutrição Departamento de Nutrição Social Rio de JaneiroRJ Brasil Universidade do Estado do Rio de Janeiro. Instituto de Nutrição. Departamento de Nutrição Social. Rio de Janeiro, RJ, Brasil

**Keywords:** Child, Food Consumption, Industrialized Foods, Food Labeling, Nutritional Value

## Abstract

**OBJECTIVE:**

To analyze the nutritional composition of ultra-processed foods consumed by children that attend basic health units.

**METHODS:**

This is a cross-sectional study with a representative probabilistic sample of 536 children aged between 6 and 59 months treated at a health unit in the city of Rio de Janeiro. Nutritional information was extracted from labels of the ultra-processed foods referred to in a 24-hour recall. The 351 foods mentioned were divided into 22 groups and 38 subgroups according to the type of product, and they were characterized according to the averages of the values for energy, total fats, saturated fats, trans fats and sodium in 100 grams of the product, in addition to presence, number, and type of “other sweeteners”. The nutritional Profile Model of the Pan American Health Organization was applied for each food and for the average of nutrient content obtained for each group to examine the occurrence of critical nutrients excess.

**RESULTS:**

Ultra-processed foods contained high energy value and high levels of total fats, saturated fats, trans fats, and sodium. Out of the total of ultra-processed foods, 66% presented excess of at least one critical nutrient, with emphasis on *requeijões* and ultra-processed cheeses, instant noodles, and industrialized and sausage-like meats, which presented 100% of foods with excess of total fats, saturated fats and sodium. Out of the 21 groups, the following exceeded the limit established by the Pan American Health Organization: for total fats, 10 groups; for saturated fats, 11; for trans fats, 3; and sodium, 13. *Requeijões* and ultra-processed cheeses; industrialized and sausage-like meats; and biscuits exceeded this limit in all parameters. Out of the set of ultra-processed foods analyzed, 13.4% contained “other sweeteners” (eight different types).

**CONCLUSIONS:**

The ultra-processed foods analyzed presented unbalanced nutritional profile, and two thirds presented excess of at least one critical nutrient. Educational actions and regulatory measures are necessary to better inform the population and to discourage its consumption.

## INTRODUCTION

Ultra-processed foods (UPF) are industrial formulations typically composed of five or more ingredients, including substances and additives^1^. Their manufacture is usually made by large industries, involving various stages and industrial processes (e.g., cornflour extrusion to produce snacks) that do not have domestic equivalents^1^. The main purpose of ultra-processing is to create ready-to-eat, drink or heat (such as sandwich biscuits and instant noodles), which can replace natural (such as leaves and fruits) or minimally processed foods (such as dried, polished and packaged grains)^1^.

Often, UPF present inadequate and unfavorable nutritional profile for health and negatively affect the nutritional quality of food, generally with high energy density and excess of total fats, saturated fats, sugar and sodium, as well as low fiber content^1,2^. However, in recent decades, UPF consumption has been increasing both worldwide and in Brazil, including among the infant population^3^, and it has been associated with negative health outcomes among children (e.g., asthma, lipid profile change and greater waist circumference), adolescents (e.g., higher body mass and body fat) and adults (e.g., obesity, cardiovascular diseases, cancer, depression, gastrointestinal disorders and higher mortality)^6^.

In 2014, the Pan American Health Organization (PAHO) developed a Nutritional Profile Model (NPM/PAHO) to classify UPF according to content of free sugars, salt, total fats, saturated fats and trans-fatty acids (called “critical nutrients”) and presence of sweeteners^7^. The objective of this model was to support regulatory measures within the framework of public policies for overweight prevention and control.

Studies examining the nutritional composition of UPF available on the market^8,9^ consumed by children and other population groups^2,5^ are described in the literature. However, in Brazil, the number of analyses of UPF nutritional quality consumed by children based on PAHO Nutritional Profile Model^7^is insignificant.

The (re)formulation of food guidance instruments and strategies can be supported by knowing the UPF consumed by children, as well as the implementation of regulatory measures and other initiatives to promote healthy eating within the framework of public policies. This study aimed to evaluate UPF consumed by children attending basic health units (BHU) in the city of Rio de Janeiro to expand the body of evidence on these foods in infant feeding. UPF were characterized according to energy value, macronutrients, sodium, and presence of natural or artificial sweeteners, and they were assessed according to the guidelines of the Nutritional Profile Model of the Pan American Health Organization.

## METHODS

This study is part of the research project *Alimentação e nutrição de pré-escolares usuários do Sistema Único de Saúde na cidade do Rio de Janeiro* (Food and nutrition of preschoolers attending the Unified Health System in the city of Rio de Janeiro), which consisted of a cross-sectional study with a probabilistic sample of children of both genders, aged between 6 and 59 months, representative of the population of this age group using the municipal basic health network in the city of Rio de Janeiro. The detailed description of this study and sampling process are available in Carneiro’s work^10^. The list of UPF consumed by the children studied composed the database for our study.

The first stage of data collection occurred in 2014, being composed of a 24-hour recall, available as supplementary material of this article, in the BHU drawn with the mother or guardian of the child on a previously scheduled day (between Tuesday and Friday). Types of food, quantities, preparation method, time, place of consumption and, in case of processed foods and UPF, their respective brands and flavors were recorded. This activity was developed by nutritionists who previously received 18-hour theoretical and practical training on how to approach the interviewee and how to fill out the 24-hour recall form. Data regarding socioeconomic and demographic characterization of the studied group and attendance at daycare or school were also collected by the application of a closed questionnaire.

The second stage, which occurred in 2015, included the identification of the UPF^1^ referred to by the study participants and market research to register their labels (including brands and flavors). In the UPF identification, if two informants referred to the same product (of the same brand and flavor), the food was computed only once. Therefore, identified UPF express the list of UPF referred to, that is, the types of product, their brands and flavors, and not the frequency of children who reported consuming each of them.

For market research, establishments from different neighborhoods were visited, and all labels were photographed. For UPF not found in establishments, label information was collected on the manufacturer’s website. When this information was not available, contact was established with the customer service, by e-mail or telephone call.

In the case of the UPF with no reference to brand by any of the interviewees, the nutritional information available in the nutritional composition table of the Household Budget Survey^11^was adopted. Such foods were: sausages, *mortadella*, boiled ham, turkey blanquet, caramel syrup, ice pops of different flavors, ketchup, and mayonnaise. The data collected on labels (nutritional information, ingredients, and others that are beyond this study scope) were typed in the software Microsoft Excel^®^ 2010.

UPF were grouped according to the type of product (e.g., sweetened beverages, biscuits, sweets and candies). Foods originally allocated in the same group but with very distinct nutritional composition were divided into subgroups (Table 1).

**Table 1:** Mean values and standard deviation of energy, total fats, saturated fat, trans fat and sodium in 100 grams of product according to groups and subgroups of the cast of ultra-processed foods consumed by children aged from 6 to 59 months attended in basic health units. City of Rio de Janeiro, 2014.

Ultra-processed foods	N^a^	Energy (kcal)	Total fats (g)	Saturated fat (g)	Trans fat (g)	Sodium (mg)
Sweetened beverages	82	33.1 (17.6)	0.0 (0.1)	0.0 (0.0)	0.00 (0.00)	7.6 (7.5)
Fruit nectar	27	47.6	0.0	0.0	0.00	4.0
Powdered drink mix	24	14.1	0.0	0.0	0.00	12.4
Soft drinks	12	42.5	0.0	0.0	0.00	5.2
Flavored beverages based on soy extract	10	33.1	0.0	0.0	0.00	0.0
Guarana and blackcurrant syrups	5	36.2	0.0	0.0	0.00	0.7
Natural guarana	4	4.2	0.0	0.0	0.00	1.5
Biscuits	60	444.3 (39.3)	16.4 (4.1)	6.5 (3.1)	0.58 (1.30)	430.9 (232.68)
Sandwich biscuits	22	463.3	19.4	8.8	0.45	228.0
Crackers	22	434.1	15.2	5.6	0.15	666.7
Biscuits	15	432.7	13.8	4.8	1.13	349.1
*Polvilho* snack	1	423.3	16.7	2.7	6.67	933.3
Sweets and candies	52	298.9 (189.0)	9.1 (12.6)	4.1 (6.5)	0.29 (1.21)	84.3 (145.70)
Candies and lollipops	15	396.3	5.5	1.8	0.96	127.0
Chocolates	10	529.0	29.3	14.7	0.00	105.9
Gelatin	10	23.7	0.0	0.0	0.00	66.8
Jams	4	126.3	0.0	0.0	0.00	22.6
Ice cream	4	177.0	5.6	3.2	0.13	93.0
Other sweets^b^	4	421.3	18.1	5.6	0.00	87.5
Bubble/chewing gums	2	244.0	0.0	0.0	0.00	0.0
Syrups and similar	2	314.5	0.8	2.7	0.16	0.0
Ice popsicle	1	79.0	0.2	0.0	0.00	7.0
Ultra-processed yogurts and dairy drinks	35	97.1 (24.0)	2.5 (1.8)	1.5 (1.2)	0.00 (0.00)	47.2 (18.19)
Ultra-processed yogurts	15	105.5	3.3	2.1	0.00	44.9
Petit suisse	8	111.9	3.1	1.7	0.00	50.0
Fermented milks	7	68.9	0.2	0.2	0.00	36.0
UHT Dairy	3	85.7	2.4	1.0	0.00	65.0
Dairy drinks with cereals	2	90.8	2.6	0.5	0.00	65.8
Snacks and potato chips	14	497.4 (35.0)	25.6 (8.2)	5.7 (3.9)	0.00 (0.00)	740.0 (259.43)
Corn or wheat snacks	12	487.3	23.6	4.9	0.00	788.3
Potato chips	2	558.0	37.6	10.4	0.00	450.0
Flours and thickeners	14	370.0 (14.8)	0.8 (1.8)	0.3 (0.8)	0.00 (0.00)	108.2 (95.85)
Growing-up milks and infant formulas	11	489.9 (521)	23.3 (3.2)	8.7 (6)	0.00 (0.00)	202.5 (57.64)
Infant formulas	9	498.2	23.9	9.1	0.00	199.1
Growing-up milks	2	452.8	20.3	7.0	0.00	217.7
Industrialized and sausage-like meats	11	240.6 (76.5)	16.5 (10.3)	5.9 (3.8)	0.39 (0.77)	773.2 (269.13)
Industrialized meats	6	211.7	11.4	4.1	0.00	590.1
Sausage-like meats	5	275.2	22.7	8.1	0.85	992.9
Ready-made seasonings in cubes or powder	11	186.7 (99.2)	8.0 (11.2)	5.4 (7.5)	0.00 (0.00)	21964.1 (5721.47)
Juice concentrate	7	4.17 (3.2)	0.0 (0.0)	0.0 (0.0)	0.00 (0.00)	1.5 (1.11)
*Requeijão* and ultra-processed cheeses	7	241.4 (57.3)	21.6 (6.5)	13.1 (4.1)	0.33 (0.58)	601.0 (131.37)
*Requeijão*	5	226.0	19.8	12.1	0.27	598.0
Ultra-processed cheeses	1	280.0	25.0	14.3	1.00	806.7
Cream cheese	1	280.0	27.0	17.0	0.00	410.0
Industrialized cakes	7	325.1 (80.0)	10.3 (5.1)	4.5 (2.7)	0.17 (0.44)	219.8 (29.30)
Cocoa powder and strawberry-based flavorings	6	386.4 (11.9)	1.0 (1.1)	0.3 (0.4)	0.00 (0.00)	188.6 (117.04)
Industrialized sauces and tomato sauces	6	85.8 (89.1)	4.2 (9.4)	0.6 (1.5)	0.02 (0.05)	669.8 (255.85)
Tomato sauce	4	39.2	0.4	0.0	0.00	518.3
Industrialized sauce	2	179.0	11.8	1.9	0.06	972.7
Bread	6	295.0 (44.5)	3.3 (1.9)	0.9 (0.7)	0.00 (0.00)	412.0 (60.37)
Small roll	3	304.7	4.5	1.3	0.00	364.0
Sliced bread	2	246.0	3.0	0.7	0.00	434.0
Toast	1	364.0	0.0	0.0	0.00	512.0
Dietary supplements	5	397.6 (36.6)	5.0 (7.5)	1.7 (1.8)	0.00 (0.00)	196.9 (94.18)
Margarine	4	597.5 (189.8)	66.3 (21.4)	17.3 (5.5)	0.00 (0.00)	612.5 (62.92)
Breakfast cereals	4	379.2 (15.5)	2.7 (2.0)	0.8 (0.9)	0.00 (0.00)	362.5 (89.87)
Industrialized *farofa*	3	391.0 (20.5)	9.6 (1.7)	3.6 (2.4)	0.00 (0.00)	486.2 (52.77)
Soy-based beverages	2	37.5 (4.2)	1.6 (0.0)	0.2 (0.0)	0.00 (0.00)	82.8 (0.35)
Instant noodles	2	440.6 (9.2)	18.2 (0.8)	8.2 (0.1)	0.00 (0.00)	1816.5 (104.82)
Industrialized popcorn	2	418.0 (25.5)	19.8 (2.5)	9.4 (2.0)	0.00 (0.00)	638.0 (494.97)

^a^ Number of ultra-processed foods reported by interviewees.

^b^ Condensed milk, chocolate flavored condensed milk, hazelnut cocoa spread and *paçoca*.

Note: The values in parentheses are the standard deviation of the group mean.

UPF characterization was composed of the description of energy value, sodium, total fats, saturated fats and trans fats per 100 grams of the product, as well as the presence and number of food additives that provide sweet flavor to food, here called “other sweeteners” (aligned to PAHO nomenclature), according to group and subgroup. For the identification of “other sweeteners,” we adopted the definition of NPM/PAHO^7^: non-caloric artificial or natural sweeteners or caloric sweeteners (polyols), not being considered fruit juices, honey or other food ingredients that can be used to sweeten. The sweeteners identified on UPF labels mentioned were grouped into: artificial non-caloric sweeteners (aspartame, sucralose, saccharin, potassium acesulfame, sodium cyclamate, sodium saccharin, and fructooligosaccharides), natural non-caloric sweeteners (stevia) and caloric sweeteners such as polyols (sorbitol, manitol, lactitol, and isomalt)^7,12,13^.

To describe the nutritional composition of the list of UPF mentioned, average energy values, total fats, saturated fats, trans fats and sodium per 100 grams or 100 milliliters of the product were produced for each group and subgroup. In relation to “other sweeteners,” the number of products with these ingredients, minimum and maximum number of “other sweeteners” types per product and, for each group, the types of sweeteners identified were described according to group and subgroup of UPF. To identify whether the UPF reported had critical nutrients excess, the NPM/PAHO^7^was used, considering the following parameters: presence of “other sweeteners” and, in 100 grams or 100 milliliters of food: total fats ≥ 30% of total energy, saturated fats ≥ 10% of total energy, trans fats ≥ 1% of total energy and sodium ≥ 1 mg/kcal.

For each food group, the percentage of UPF exceeding the limit defined by PAHO for critical nutrients or presented sweeteners in the list of ingredients was calculated. Additionally, PAHO parameters were also applied to mean values of energy percentages from total fat, saturated fat, trans fat and to the mean sodium value, in milligrams per calorie, observed in each group in order to produce a synthesis on the quality of the UPF groups in relation to some of the critical nutrients. These results were presented as graphs.

Foods and beverages for particular purposes, such as infant formulae and dietary supplements, were excluded from the analysis according to the MPN/PAHO since they are subject to specific regulations. Furthermore, some ready-made seasonings did not contain calories, and it was not possible to evaluate the sodium content proposed in the model. For these reasons, these products were not considered in this analysis based on NPM/PAHO.

According to current Brazilian legislation, the presence of information on some micronutrients and simple sugar is not mandatory on the product label^14^. For this reason, and considering the heterogeneity of the availability of this information in the labels studied, it was not possible to describe it or its analysis according to NPM/PAHO.

Software Statistical Package for the Social Sciences^®^ (SPSS) version 19 and Microsoft Excel^®^ 2010 were used to perform the analytical procedures. The main study from which we extracted the data analyzed was approved by the Rio de Janeiro Municipal Health Office Ethics Committee for Research with Humans (process No. 93/13), and it had financial support of CNPq (processes no. 480804/2013-3 and 420247/2016-5) and the Municipal Health Department of Rio de Janeiro.

## RESULTS

The group in which the information on UPF consumption was collected was composed of 536 children. Out of these, 49.8% were girls and 54.3% attended daycare centers. Regarding maternal schooling level, 20.4% had some elementary school; 34.2%, elementary school; 42%, high school; and 3.32%, college. Regarding monthly family income, 15% of families received less than one minimum wage (R$ 724.00 at the time); 63.9%, between one and two; and 15.7%, three or more.

For nine of the products mentioned, interviewees did not mention brands, or the brand mentioned was not found in the city markets or was neither available on the manufacturer’s web page nor in the customer service. A total of 351 UPF were studied, and 335 that were not infant formulas, dietary supplements, or ready-made seasonings without calories were included in the analysis based on the MPN/PAHO. The ultra-processed foods of different brands and flavors mentioned by the interviewees were divided into 22 groups and 38 subgroups. Among these, the number of types of foods mentioned stand out: sweetened beverages (n = 82); biscuits (n = 60); sweets and candies (n = 52); ultra-processed yogurts and dairy beverages (n = 35); snacks and potato chips (n = 14); and flours and thickeners (n = 14) (Table 1).

Regarding nutritional composition, the UPF groups with the highest energy value (in 100 grams) were: margarines (597.5 kcal); snacks and potato chips (497.4 kcal); growing-up milks and infant formulae (not reconstituted; 489.9 kcal,); biscuits (444.3 kcal); instant noodles (440.6 kcal); and industrialized popcorn (418.0 kcal). Considering total fats (in 100 grams), the groups with the highest levels were: margarines (66.3 g); snacks and potato chips (25.6 g); growing-up milks and infant formulae (not reconstituted; 23.3 g); industrialized popcorn (19.8 g); instant noodles (18.2 g); and biscuits (16.4 g). The groups with the highest levels of saturated fat (in 100 g) were: margarines (17.0 g); industrialized popcorn (9.4 g); instant noodles (8.2 g); biscuits (6.5 g); snacks and potato chips (5.7 g); and ready-made seasonings (5.4 g). Trans fats were observed in higher amounts (in 100 g) in the groups: biscuits (0.58 g); industrialized and sausage-like meat (0.39 g); *requeijões* and ultra-processed cheeses (0.33 g); and sweets and candies (0.29 g). The highest sodium levels (in 100 g) were observed in the groups: ready-made seasonings (21,964.1 mg); instant noodles (1,816.5 mg); industrialized meat (773.2 mg); snacks and potato chips (740.0 mg); and industrialized sauces and tomato sauces (669.8 mg), as shown in Table 1.

Among groups and subgroups presenting UPF with “other sweeteners” in their lists of ingredients, it is worth mentioning: sweetened beverages (out of the 82 UPF, 26 [32%] contained sweeteners, with emphasis on powdered drink mix and soy-based flavored beverages); sweets and candies (11 [21%] of the 52 UPF, especially gelatin); and growing-up milks and infant formulas (3 [27%] of the 11 UPF, with emphasis on growing-up milks). These results are presented in Table 2. Note that, of the 46 foods presenting “other sweeteners,” three were intended for special diets.

**Table 2:** Frequency and type of “other sweeteners” present in the lists of ingredients of the ultra-processed foods (UPF) that contained sweeteners consumed by children aged from 6 to 59 months attending basic health units. City of Rio de Janeiro, 2014.

Ultra-processed foods^a^	UPF number^b^ with “other sweeteners” (% of total foods mentioned)	UPF number according to number of “other sweeteners” present						Types of “other sweeteners” present in each group and UPF number in which each type of “other sweetener” was present
		1	2	3	4	5	6	
Sweetened beverages	**26 (31.7)**	**6**	**7**	**1**	**12**	**0**	**0**	Non-caloric artificial (n): acesulfame potassium (20), sodium cyclamate (18), aspartame (14), sodium saccharin (13), sucralose (6)
Powdered drink mix	20 (83.3)	0	7	1	12	0	0	
Flavored beverages based on soy extract	6 (60.0)	6	0	0	0	0	0	
Biscuits	**1 (1.7)**	**1**	**0**	**0**	**0**	**0**	**0**	Caloric natural (poliyols): sorbitol (1)
Sandwich biscuits	1 (4.5)	1	0	0	0	0	0	
Sweets and candies	**11 (21.2)**	**1**	**1**	**2**	**6**	**0**	**1**	Non-caloric artificial: sodium cyclamate (10), aspartame (9), sodium saccharin (9), acesulfame potassium (7), sucralose (1), Caloric natural (polyols): sorbitol (1), manitol (1)
Gelatin	10 (100.0)	1	1	2	6	0	0	
Bubble/chewing gums	1 (50.0)	0	0	0	0	0	1	
Ultra-processed yogurts and dairy drinks	**1 (2.9)**	**0**	**0**	**0**	**0**	**0**	**0**	Non-caloric artifical: sucralose (1)
Fermented milks	1 (14.3)	0	0	0	0	0	0	
Growing-up milks and infant formulae	**3 (27.3)**	**3**	**0**	**0**	**0**	**0**	**0**	Non-caloric artificial fructooligosaccharides (3)
Infant formulae	1(11.1)	1	0	0	0	0	0	
Modified growing-up milks	2 (100.0)	3	0	0	0	0	0	
Industrialized cakes	**2 (28.6)**	**2**	**0**	**0**	**0**	**0**	**0**	Caloric natural (poliyols): sorbitol (2)
Soy-based beverages	**2 (100.0)**	**2**	**0**	**0**	**0**	**0**	**0**	Non-caloric artificial: sucralose (2)

^a^ Only groups and subgroups that have “other sweeteners” in the ingredient list.

^b^ Number of ultra-processed foods reported by interviewees.

Regarding the types of “other sweeteners” present in the analyzed groups, we observed that 26 sweetened beverages in which these ingredients were identified presented one to four different types of “other sweeteners” in a single product. All “other sweeteners” identified in sweetened beverages were non-caloric artificial: acesulfame potassium, sodium cyclamate, aspartame, sodium saccharin, and sucralose. In the group of sweets and candies, six different types of “other sweeteners” were identified in a single product. Notably, the 10 gelatins analyzed contained “other sweeteners,” and 60% (six) had four different types. Among the 11 foods of the sweets and candies group containing “other sweeteners,” non-caloric artificial sweeteners were identified − sodium cyclamate, aspartame, sodium saccharin, acesulfame potassium and sucralose – and natural caloric sweeteners (polyols) − sorbitol and mannitol (Table 2).

Out of the 335 UPF analyzed individually according to the MPN/PAHO, 66% presented “other sweeteners” or excess of at least one critical nutrient. Out of the total foods, 32.5% presented excess total fats; 36.4%, of saturated fats; 36.7%, sodium; 7%, trans fats; and 13.4% presented “other sweeteners” in their ingredients. In 13 of the 21 groups included in this analysis, all UPF presented excess of at least one critical nutrient. They were: soy-based beverages; snacks and potato chips; ultra-processed *requeijões* and ultra-processed cheeses; margarines; growing-up milks; industrialized and sausage-like meats; ready-made seasonings; processed sauces and tomato sauces; bread; industrialized cakes; industrialized *farofa*; instant noodles; and industrialized popcorn. The groups of *requeijões* and ultra-processed cheeses, instant noodles, and industrialized and sausage-like meats presented 100% of foods with excess total fats, saturated fats, and sodium. UPF groups that did not present excess of any of the critical nutrients analyzed were: juice concentrate; flours and thickeners; cocoa powder, and strawberry flavoring (Table 3).

**Table 3:** Percentage of ultra-processed foods (UPF) that presented excess of total fats, saturated fats, trans fats, and sodium in 100 grams of the product and presence of “other sweeteners”a according to groups of ultra-processed foods consumed by children aged from 6 to 59 months attending basic health units. City of Rio de Janeiro, 2014.

Ultra-processed food groups	N^b^	% of UPF with excess critical nutrient^c^					% of UPF with at least one critical nutrient in excess and/or presence of “other sweeteners”
		Total fats	Saturated fat	Trans fat	Sodium	Presence of “other sweeteners”	
Sweetened beverages	82	0.0	0.0	0.0	25.6	31.7	35.4
Biscuits	60	61.7	68.3	23.3	46.7	1.7	95.0
Sweets and candies	52	28.8	34.6	9.6	21.2	21.2	65.4
Ultra-processed yogurts and dairy drinks	35	14.3	65.7	0.0	0.0	2.9	65.7
Snacks and potato chips	14	92.9	14.3	0.0	78.6	0.0	100.0
Flours and thickeners	14	0.0	0.0	0.0	0.0	0.0	0.0
Industrialized and sausage-like meats	11	100.0	100.0	27.3	100.0	0.0	100.0
Ready-made seasonings	9	44.4	44.4	0.0	100.0	0.0	100.0
Juice concentrate	7	0.0	0.0	0.0	0.0	0.0	0.0
*Requeijão* and ultra-processed cheeses	7	100.0	100.0	28.6	100.0	0.0	100.0
Industrialized cakes	7	42.9	57.1	14.3	14.3	28.6	100.0
Cocoa powder and strawberry-based flavorings	6	0.0	0.0	0.0	0.0	0.0	0.0
Industrialized sauces and tomato sauces	6	33.3	16.7	0.0	100.0	0.0	100.0
Bread	6	0.0	0.0	0.0	100.0	0.0	100.0
Margarine	4	100.0	100.0	0.0	25.0	0.0	100.0
Breakfast cereals	4	0.0	0.0	0.0	75.0	0.0	75.0
Industrialized *farofa*	3	0..0	33.3	0.0	100.0	0.0	100.0
Soy-based beverages	2	100.0	0.0	0.0	100.0	100.0	100.0
Growing-up milks	2	100.0	100.0	0.0	0.0	100.0	100.0
Instant noodles	2	100.0	100.0	0.0	100.0	0.0	100.0
Industrialized popcorn	2	100.0	100.0	0.0	50.0	0.0	100.0
Total	335	32.5	36.4	7.5	36.7	13.4	66.0

^a^ According to PAHO’s Nutritional Profile Model^7^.

^b^ Number of UPF reported by interviewees.

^c^ Sugars were not considered in this classification.

**Figure 1:**
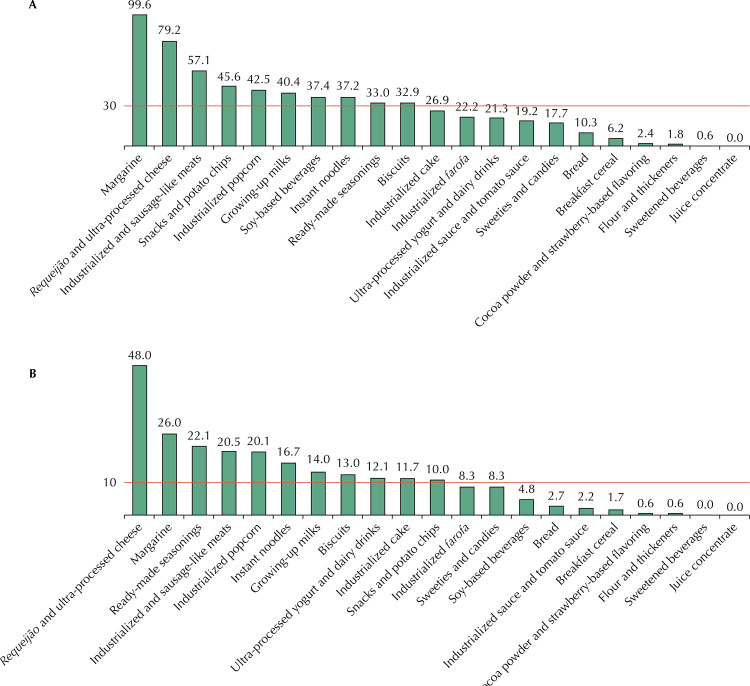
Average percentages of energy from total fat (A) and saturated fat (B) in 100 grams of product per group of ultra-processed foods consumed by children aged 6 to 59 months attending basic health units compared with the line representing PAHO’s recommendation*. City of Rio de Janeiro, 2014. * PAHO Nutrient Nutritional Profile Model^7^, which considers total fats excess, whether in a certain amount of food, the amount of energy (kcal) from total fats (grams of total fats × 9 kcal) is equal to 30% or more of the total energy value (kcal) and excess saturated fats if, in a given amount of food, the amount of energy (kcal) derived from saturated fats (grams of saturated fats × 9 kcal) is equal to 10% or more of the total energy value (kcal).

**Figure 2:**
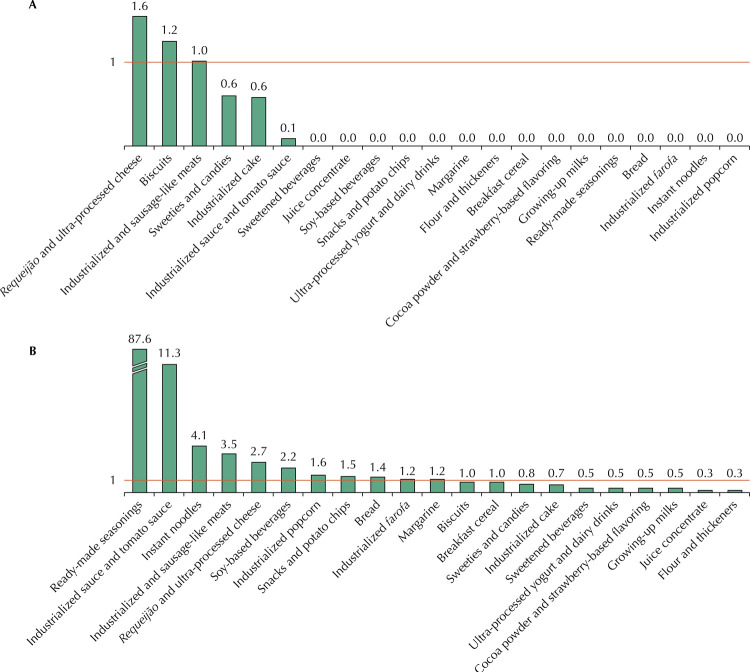
Average percentage of energy from trans fat (A) and ratio between amount of sodium (mg) and energy value (kcal) (B) in 100 grams of product per group of ultra-processed foods consumed by children aged 6 to59 months attending basic health units with a line representing PAHO’s recommendation*. City of Rio de Janeiro, 2014. * PAHO Nutritional Profile Model^7^, which considers trans fats excess if, in a given amount of food, the amount of energy (kcal) from trans fats (grams of trans fats × 9 kcal) is equal to 1% or more of the total energy value (kcal) and excess sodium when the ratio between the amount of sodium (mg) in a given amount of food and the energy value (kcal) is 1:1 or greater.

The application of PAHO parameters to mean values, in each group of UPF, of the percentages of energy from total fat, saturated fat and trans fat, as well as to the mean sodium value in milligrams per kilocalories, showed that, out of the 21 groups analyzed, 10 groups exceeded the limit established for total fats; 11 for saturated fats; 3 for trans fats; and 13 for sodium (Figures 1 and 2). Notably, many UPF groups exceeded the limit established by PAHO for total fats, saturated fats, and sodium, and some of them (biscuits, *requeijões* and ultra-processed cheeses, industrialized and sausage-like meats) exceeded the limits established by PAHO in all parameters.

## DISCUSSION

Most UPF reported by the participants are nutritionally unbalanced since they contain high energy value or high content of total fats, saturated fats, trans fats, and sodium. When analyzed according to the MPN/PAHO, most of them presented excess of at least one of the critical nutrients or presence of “other sweeteners” (from one to six types).

These results corroborate other studies that indicate these foods present unfavorable nutritional profile^15,16^. However, a more in-depth comparison of our results with the available literature is not a simple task, considering the methodological differences between studies, either concerning the classification of foods (not based on the extent and purpose of their processing^17^), the parameters for assessing their nutritional profile (criteria different from those of PAHO to establish limits for critical nutrients^18^), the age group considered (under two years^17^ or children and not children^19^), the analysis approach (e.g., comparison of the nutritional composition of products aimed to children, with and without health claim^9^), or empirical basis (e.g., products available on market^8^).

Considering these differences, other studies indicate results that converge with those found here. In Canada, in 2005, an analysis of the nutritional profile of products targeted to children in a supermarket found that, out of the 327 products analyzed, 89% could be classified as poor nutritional quality (high amounts of sugar, fat, or sodium)^18^. A similar result was observed in a study conducted in Australia in 2009: out of the 157 products marketed to children assessed by the study, 75.2% presented a high concentration of fat or sugar^20^.

Studies comparing UPF aimed to the infant and non-infant group showed that the former did not present a more favorable nutritional composition. A survey conducted in 2013 in Florianópolis compared the products directed to these two groups and identified that chocolate, sandwich, biscuits and cookies, candies, soft drinks and snacks directed to children presented lower amount of total fats, lower amount of fiber and higher amount of sodium than those directed to other age groups^21^. A study conducted in the United Kingdom between 2010 and 2011 showed that a significant number of products marketed to children had higher levels of fat, sugar, and salt than those marketed to the general population^19^.

In PAHO document, which proposes criteria for critical nutrient analysis, the nutritional profile of some UPF directed to audiences from different countries^7^was compared. In the case of Brazil, UPF groups in which 100% of the analyzed products presented excessive levels of one or more critical nutrients were: breakfast cereals; packaged bread; sugared yogurt; sugared milk; ice cream; hams; caramels and chocolates; and sugar-sweetened beverages^7^. Data were similar to our results regarding excessive content of at least one critical nutrient in all packaged bread, hams, sugared milks and caramels, and chocolates analyzed.

Because the MPN/PAHO has been recently published, there are still few studies applying the parameters proposed by it. In a survey conducted in 2014 in Uruguay in which these parameters were used, it was observed that all ultra-processed foods directed to children available in supermarkets in Montevideo had excessive levels of at least one critical nutrient^8^. Comparing with the results of our study, the group of biscuits and cakes and the dairy group presented a worse nutritional profile in that country^8^. In Argentina, a study conducted in 2014 that evaluated the number of ads for processed and ultra-processed foods targeted to children on open television channels found that in 2,959 ads, 93.1% advertised products had excessive energy levels and/or one or more critical nutrients^22^. In 2017, a Canadian study found that out of 365 processed and ultra-processed products aimed at children available in a supermarket, 86.5% had free sugars excess; 36.2%, sodium excess; 32.5%, saturated fat excess; and 28.7%, total fat excess^23^.

The results on the nutritional composition of UPF are worrisome because of their relation to unfavorable health outcomes^6^. Moreover, sales and consumption of these UPF have been increasing worldwide recently, with recurrent consumption among children^3,4,5^.

Regarding sweeteners, the habitual consumption of sweet-tasting products by children has been associated with a lower quality of diet, defining lifetime consumption patterns^24^. Furthermore, some studies suggest that early exposure to sweeteners (caloric and non-caloric sweeteners) may adversely affect body composition, cardiometabolic health, and intestinal microbiota^24,25^.

This study presents some limitations. Some interviewees did not mention the brands of the products consumed or mentioned brands that were not found in the city’s markets or were not available on the manufacturer’s web page or in the customer service. These factors may have led to underestimation of the list of analyzed products and, therefore, errors (for more or less) in the nutritional composition of the UPF groups analyzed.

Also, the fact that characteristics of the labeling legislation in Brazil have limited the analyses related to sugars. As this information is not mandatory^14^, many labels do not disclose it. This prevented the analysis of the parameter “free sugars excess,” provided for in the NPM/PAHO. The consequence of this was non-characterization of this parameter for UPF groups frequently mentioned by the studied group and in which sugar is one of the main ingredients: breakfast cereals; industrialized cakes; ultra-processed yogurts and dairy beverages; sweets and candies; sweetened beverages; flours and thickeners; and cocoa powder and strawberry-based flavorings. Moreover, in current Brazilian legislation, mandatory nutritional labeling on trans fat refers to portion of the product (not 100 g), and those with levels inferior to or equal to 0.2 g per portion do not need to report the presence of this nutrient^14^. Thus, the presence and content of trans fat in the set of products mentioned by the participants may have been underestimated. That is, the nutritional profile of UPF reported by the study group may be even less adequate than that described here.

The data analyzed originate from only one 24-hour recall. This procedure is appropriate since the aim of this study was to analyze the nutritional composition of foods mentioned by the participants and not to quantify, at individual level, the consumption of macro and micronutrients and to associate it with other events of interest, a purpose that could justify the performance of at least two 24-hour recall^26^. As the interviews occurred between Tuesday and Friday, they provided a conservative overview of children’s feeding, since the consumption of ultra-processed foods on weekends may be even greater.

Among the strengths of the study, it is worth mentioning that the products analyzed are those mentioned by a representative sample of children under five years of age who are attended by SUS in a large Brazilian city. This choice offers two advantages over the most recurrent approach in the literature, the analysis of products available in the local markets^8,18,19,21^. The first is to support public policies directed to this group to be implemented in the public primary care network, since it enables the characterization of UPF effectively consumed by this group. The second advantage is to provide an overview of which ultra-processed foods are actually consumed by children, regardless of whether they are targeted to this audience, which can support broader regulatory measures, such as regulation on price, labeling, and advertising of these foods.

Another aspect to be valued is the analysis performed based on NPM/PAHO^7^, still incipient in the literature^8,22,23^ and, as far as we know, a pioneer in Brazil. Also on this analysis, it is noteworthy the exercise of applying PAHO parameters to the average percentage of critical nutrients examined. This enabled an interpretation complementary to that recommended by PAHO, which provides an overview of food groups in relation to these parameters.

In conclusion, this study showed that ultra-processed foods consumed by children that attend the Unified Health System in the city of Rio de Janeiro has inadequate nutritional profile and most of them present excessive levels of at least one critical nutrient for public health problems. Furthermore, a portion of them contains at least one “other sweetener.” This evidence suggests that, in addition to educational actions that stimulate the preservation of Brazilian food culture, to decrease the consumption of ultra-processed foods, it is urgent to disseminate the Dietary Guidelines of the Brazilian Population, which recommends avoiding these foods, as well as the implementation of regulatory measures that: (a) restrict market practices that encourage their consumption; (b) tax the UPF, increasing their final price; (c) improve food labeling, with more transparent nutritional information and frontal labeling warning for critical nutrients and sweeteners, in the light of what has been discussed under ANVISA^27^; and (d) restrict the supply of UPF and expand the supply of natural food or minimally processed in the school environment.

Future studies are necessary to expand the evidence on ultra-processed foods, their ingredients, their consumption in different population groups, their relation to health outcomes, and analyze them based on the NPM/PAHO perspective.
