# Supplementary material for: Nutritional profile of ultra-processed foods consumed by children in Rio de Janeiro
Source: Rev Saude Publica. 2020 Aug 28;54:89. doi: 10.11606/s1518-8787.2020054001752 (PMC7454167; doi:10.11606/s1518-8787.2020054001752)
Supplement: Translation

# Translation: Perfil nutricional de alimentos ultraprocessados consumidos por crianças no Rio de Janeiro

**Authors:** Carine de Oliveira Avelar Anastácio, Juliana Martins Oliveira, Milena Miranda de Moraes, Jorginete de Jesus Damião, Inês Rugani Ribeiro de Castro

**Affiliations:** I Brasil Universidade do Estado do Rio de Janeiro. Instituto de Nutrição. Programa de Pós-Graduação em Alimentação, Nutrição e Saúde. Rio de Janeiro, RJ, Brasil; II Brasil Universidade do Estado do Rio de Janeiro. Instituto de Nutrição. Departamento de Nutrição Social. Rio de Janeiro, RJ, Brasil

**Keywords:** Criança, Consumo de Alimentos, Alimentos Industrializados, Rotulagem de Alimentos, Valor Nutritivo

## Abstract

**OBJETIVO:**

Analisar a composição nutricional dos alimentos ultraprocessados consumidos por crianças usuárias de unidades básicas de saúde.

**MÉTODOS:**

Trata-se de estudo seccional com amostra probabilística representativa de 536 crianças de 6 a 59 meses de idade atendidas em uma unidade de saúde no município do Rio de Janeiro. Informações nutricionais foram extraídas dos rótulos dos alimentos ultraprocessados referidos em recordatório de 24 horas. Os 351 alimentos citados foram divididos em 22 grupos e 38 subgrupos de acordo com o tipo de produto e caracterizados segundo as médias dos valores encontrados para energia, gorduras totais, gorduras saturadas, gorduras trans e sódio em 100 gramas do produto, além de presença, número e tipo de “outros edulcorantes”. Para examinar a ocorrência de excesso de nutrientes críticos, o Modelo de Perfil Nutricional da Organização Pan-Americana da Saúde foi aplicado para cada alimento e para a média dos teores de nutrientes obtida para cada grupo.

**RESULTADOS:**

Os alimentos ultraprocessados consumidos continham alto valor energético e elevados teores de gorduras totais, gorduras saturadas, gorduras trans e sódio. Do total de alimentos ultraprocessados, 66% apresentaram excesso de pelo menos um nutriente crítico, com destaque para requeijões e queijos ultraprocessados, macarrões instantâneos e carnes industrializadas e embutidos, que apresentaram 100% dos alimentos com excesso de gorduras totais, de gorduras saturadas e de sódio. Dos 21 grupos, extrapolaram o limite estabelecido pela Organização Pan-Americana da Saúde: para gorduras totais, 10 grupos; para gorduras saturadas, 11; para gorduras trans, 3; e para sódio, 13. Requeijões e queijos ultraprocessados; carnes industrializadas e embutidos; e biscoitos superaram esse limite em todos os parâmetros. Do conjunto de alimentos ultraprocessados analisados, 13,4% continham “outros edulcorantes” (oito diferentes tipos).

**CONCLUSÕES:**

Os AUP analisados apresentaram perfil nutricional desequilibrado e dois terços apresentaram excesso de pelo menos um nutriente crítico. Fazem-se necessárias ações educativas e medidas regulatórias para melhor informar a população e desencorajar o seu consumo.

## INTRODUÇÃO

Os alimentos ultraprocessados (AUP) são formulações industriais feitas tipicamente com cinco ou mais ingredientes, incluindo substâncias e aditivos^1^. Sua fabricação é feita em geral por indústrias de grande porte, envolvendo diversas etapas e processos industriais (por exemplo, a extrusão da farinha de milho para fazer salgadinhos “de pacote”) que não possuem equivalentes domésticos^1^. O principal propósito do ultraprocessamento é criar alimentos prontos para comer, beber ou aquecer (como biscoitos recheados e macarrão instantâneo), que sejam capazes de substituir alimentos *in natura* (como folhas e frutos) ou minimamente processados (como grãos secos, polidos e empacotados)^1^.

Frequentemente, os AUP apresentam perfil nutricional inadequado e desfavorável para a saúde e impactam negativamente a qualidade nutricional da alimentação, em geral com alta densidade energética e excesso de gorduras totais, gorduras saturadas, açúcar e sódio, além de baixo teor de fibras^1,2^. Contudo, nas últimas décadas, o consumo de AUP tem sido expressivo, vem aumentando no mundo e no Brasil, inclusive na população infantil^3^, e tem sido associado a desfechos negativos em saúde entre crianças (por exemplo, asma, alteração de perfil lipídico e maior circunferência da cintura), adolescentes (por exemplo, maior massa corporal e gordura corporal) e adultos (por exemplo, obesidade, doenças cardiovasculares, câncer, depressão, desordens gastrointestinais e maior mortalidade)^6^. A Organização Pan-Americana da Saúde (OPAS) desenvolveu, em 2014, um Modelo de Perfil Nutricional (MPN/OPAS) para classificar os AUP em função do teor de açúcares livres, sal, gorduras totais, gorduras saturadas e ácidos graxos trans (denominados “nutrientes críticos”) e da presença de edulcorantes^7^. Seu objetivo foi disponibilizar um instrumento que apoiasse medidas regulatórias no âmbito de políticas públicas para prevenção e controle do excesso de peso.

São descritos na literatura estudos que examinam a composição nutricional dos AUP disponíveis no mercado^8,9^ consumidos por crianças e outros grupos populacionais^2,5^. Contudo, é insignificante no Brasil o número de análises da qualidade nutricional dos AUP consumidos por crianças com base no Modelo de Perfil Nutricional da OPAS^7^.

Conhecer os AUP consumidos por crianças pode subsidiar a (re)formulação de instrumentos e estratégias de orientação alimentar, bem como a implementação de medidas regulatórias e outras iniciativas de promoção da alimentação saudável no âmbito das políticas públicas. Com o intuito de ampliar o corpo de evidências sobre esses alimentos na alimentação infantil, este estudo buscou avaliar os AUP consumidos por crianças usuárias de unidades básicas de saúde (UBS) da cidade do Rio de Janeiro. Os AUP foram caracterizados de acordo com o valor energético, macronutrientes, sódio e presença de edulcorantes naturais ou artificiais e avaliados de acordo com as diretrizes do Modelo de Perfil Nutricional da Organização Pan-Americana de Saúde.

## MÉTODOS

Este estudo faz parte da pesquisa “Alimentação e nutrição de pré-escolares usuários do Sistema Único de Saúde na cidade do Rio de Janeiro”, que consistiu em estudo seccional com amostra probabilística de crianças de ambos os sexos, com idade entre 6 e 59 meses, representativa da população dessa faixa etária usuária da rede básica municipal de saúde no município do Rio de Janeiro. A descrição detalhada sobre essa pesquisa e o processo de amostragem estão disponíveis no trabalho de Carneiro^10^. O elenco de AUP consumidos pelas crianças estudadas nessa pesquisa constituiu a base de dados para o presente estudo.

A primeira etapa da coleta de dados, ocorrida em 2014, consistiu na realização de um recordatório de 24 horas (R-24h), disponível como material suplementar deste artigo, nas UBS sorteadas com a mãe ou responsável pela criança em dia previamente agendado (de terça a sexta-feira). Foram registrados os tipos de alimento, quantidades, formas de preparo, hora, local do consumo e, no caso de alimentos processados e AUP, suas respectivas marcas e sabores. Essa atividade foi desenvolvida por nutricionistas que receberam previamente treinamento teórico-prático de 18 horas sobre como abordar o(a) respondente e como preencher o formulário do R-24h. Também foram coletados, por meio da aplicação de questionário fechado, dados referentes à caracterização socioeconômica e demográfica do grupo estudado e à frequência na creche ou escola.

A segunda etapa, ocorrida em 2015, abarcou a identificação dos AUP^1^ referidos pelos participantes do estudo e uma pesquisa de mercado para registro de seus rótulos (incluindo marcas e sabores). Na identificação dos AUP, se dois informantes referissem o mesmo produto (de mesma marca e sabor), o alimento era computado uma única vez. Portanto, os AUP identificados expressam o elenco de AUP referidos, isto é, os tipos de produto, suas marcas e sabores, e não a frequência de crianças que referiram consumir cada um deles.

Para a pesquisa de mercado, foram visitados estabelecimentos de diferentes bairros, sendo fotografadas todas as faces de todos os rótulos. Para os AUP não encontrados nos estabelecimentos, as informações do rótulo foram levantadas na página eletrônica do fabricante. Quando essa informação não estava disponível, foi feito contato com o serviço de atendimento ao consumidor, por e-mail ou ligação telefônica.

No caso dos nove AUP para os quais não houve referência a marcas por nenhum dos entrevistados, foram adotadas as informações nutricionais disponíveis na tabela de composição nutricional da Pesquisa de Orçamentos Familiares^11^. Tais alimentos foram: linguiça, salsicha, mortadela, presunto cozido, *blanquet* de peru, calda de caramelo, picolé de diferentes sabores, ketchup e maionese. Os dados coletados nos rótulos (informações nutricionais, ingredientes e outras que fogem ao escopo deste estudo) foram digitados no *software* Microsoft Excel^®^ 2010.

Os AUP foram reunidos em grupos de acordo com o tipo de produto (ex.: bebidas açucaradas, biscoitos, doces e guloseimas). Quando observada composição nutricional muito distinta entre alimentos originalmente alocados em um mesmo grupo, ele foi dividido em subgrupos (Tabela 1).

**Tabela 1:** Valores médios e desvio-padrão de energia, gorduras totais, gordura saturada, gordura trans e sódio em 100 gramas de produto segundo grupos e subgrupos do elenco de alimentos ultraprocessados consumidos por crianças de 6 a 59 meses de idade atendidas em unidades básicas de saúde. Município do Rio de Janeiro, 2014.

Alimentos ultraprocessados	N^a^	Energia (kcal)	Gorduras totais (g)	Gordura saturada (g)	Gordura trans (g)	Sódio (mg)
Bebidas açucaradas	82	33,1 (17,6)	0,0 (0,1)	0,0 (0,0)	0,00 (0,00)	7,6 (7,5)
Néctares de frutas	27	47,6	0,0	0,0	0,00	4,0
Refrescos em pó	24	14,1	0,0	0,0	0,00	12,4
Refrigerantes	12	42,5	0,0	0,0	0,00	5,2
Bebidas saborizadas à base de extrato de soja	10	33,1	0,0	0,0	0,00	0,0
Xaropes de guaraná e groselha	5	36,2	0,0	0,0	0,00	0,7
Guaranás naturais	4	4,2	0,0	0,0	0,00	1,5
Biscoitos	60	444,3 (39,3)	16,4 (4,1)	6,5 (3,1)	0,58 (1,30)	430,9 (232,7)
Biscoitos doces com recheio	22	463,3	19,4	8,8	0,45	228,0
Biscoitos salgados sem recheio	22	434,1	15,2	5,6	0,15	666,7
Biscoitos doces sem recheio	15	432,7	13,8	4,8	1,13	349,1
Biscoitos polvilho	1	423,3	16,7	2,7	6,67	933,3
Doces e guloseimas	52	298,9 (189,0)	9,1 (12,6)	4,1 (6,5)	0,29 (1,21)	84,3 (145,7)
Balas e pirulitos	15	396,3	5,5	1,8	0,96	127,0
Chocolates	10	529,0	29,3	14,7	0,00	105,9
Gelatinas	10	23,7	0,0	0,0	0,00	66,8
Geleias	4	126,3	0,0	0,0	0,00	22,6
Sorvetes	4	177,0	5,6	3,2	0,13	93,0
Outras guloseimas^b^	4	421,3	18,1	5,6	0,00	87,5
Chicletes	2	244,0	0,0	0,0	0,00	0,0
Caldas e afins	2	314,5	0,8	2,7	0,16	0,0
Picolés	1	79,0	0,2	0,0	0,00	7,0
Iogurtes ultraprocessados e bebidas lácteas	35	97,1 (24,0)	2,5 (1,8)	1,5 (1,2)	0,00 (0,00)	47,2 (18,2)
Iogurtes ultraprocessados	15	105,5	3,3	2,1	0,00	44,9
Petit suisse	8	111,9	3,1	1,7	0,00	50,0
Leites fermentados	7	68,9	0,2	0,2	0,00	36,0
Bebidas lácteas UHT	3	85,7	2,4	1,0	0,00	65,0
Bebidas lácteas com cereais	2	90,8	2,6	0,5	0,00	65,8
Salgadinhos e chips	14	497,4 (35,0)	25,6 (8,2)	5,7 (3,9)	0,00 (0,00)	740,0 (259,4)
Salgadinhos de milho ou trigo	12	487,3	23,6	4,9	0,00	788,3
Chips de batata	2	558,0	37,6	10,4	0,00	450,0
Farinhas e espessantes	14	370,0 (14,8)	0,8 (1,8)	0,3 (0,8)	0,00 (0,00)	108,2 (95,8)
Compostos lácteos e fórmulas infantis	11	489,9 (52,1)	23,3 (3,2)	8,7 (1,6)	0,00 (0,00)	202,5 (57,6)
Fórmulas infantis	9	498,2	23,9	9,1	0,00	199,1
Compostos lácteos	2	452,8	20,3	7,0	0,00	217,7
Carnes industrializadas e embutidos	11	240,6 (76,5)	16,5 (10,3)	5,9 (3,8)	0,39 (0,77)	773,2 (269,1)
Carnes industrializadas	6	211,7	11,4	4,1	0,00	590,1
Embutidos	5	275,2	22,7	8,1	0,85	992,9
Temperos prontos em cubo ou em pó	11	186,7 (99,2)	8,0 (11,2)	5,4 (7,5)	0,00 (0,00)	21.964,1 (5.721,5)
Sucos concentrados	7	4,17 (3,2)	0,0 (0,0)	0,0 (0,0)	0,00 (0,00)	1,5 (1,1)
Requeijão e queijos ultraprocessados	7	241,4 (57,3)	21,6 (6,5)	13,1 (4,1)	0,33 (0,58)	601,0 (131,4)
Requeijão	5	226,0	19,8	12,1	0,27	598,0
Queijos ultraprocessados	1	280,0	25,0	14,3	1,00	806,7
Cream cheese	1	280,0	27,0	17,0	0,00	410,0
Bolos industrializados	7	325,1 (80,0)	10,3 (5,1)	4,5 (2,7)	0,17 (0,44)	219,8 (29,3)
Achocolatados e saborizantes à base de morango	6	386,4 (11,9)	1,0 (1,1)	0,3 (0,4)	0,00 (0,00)	188,6 (117,0)
Molhos industrializados e de tomate	6	85,8 (89,1)	4,2 (9,4)	0,6 (1,5)	0,02 (0,05)	669,8 (255,8)
Molho de tomate	4	39,2	0,4	0,0	0,00	518,3
Molhos industrializados	2	179,0	11,8	1,9	0,06	972,7
Pães	6	295,0 (44,5)	3,3 (1,9)	0,9 (0,7)	0,00 (0,00)	412,0 (60,4)
Bisnaguinhas	3	304,7	4,5	1,3	0,00	364,0
Pães de forma	2	246,0	3,0	0,7	0,00	434,0
Torradas	1	364,0	0,0	0,0	0,00	512,0
Suplementos alimentares	5	397,6 (36,6)	5,0 (7,5)	1,7 (1,8)	0,00 (0,00)	196,9 (94,2)
Margarinas	4	597,5 (189,8)	66,3 (21,4)	17,3 (5,5)	0,00 (0,00)	612,5 (62,9)
Cereais matinais	4	379,2 (15,5)	2,7 (2,0)	0,8 (0,9)	0,00 (0,00)	362,5 (89,9)
Farofa industrializada	3	391,0 (20,5)	9,6 (1,7)	3,6 (2,4)	0,00 (0,00)	486,2 (52,8)
Bebidas à base de extrato de soja	2	37,5 (4,2)	1,6 (0,0)	0,2 (0,0)	0,00 (0,00)	82,8 (0,3)
Macarrões instantâneos	2	440,6 (9,2)	18,2 (0,8)	8,2 (0,1)	0,00 (0,00)	1.816,5 (104,8)
Pipocas industrializadas	2	418,0 (25,5)	19,8 (2,5)	9,4 (2,0)	0,00 (0,00)	638,0 (495,0)

^a^ Número de alimentos ultraprocessados referidos pelos entrevistados.

^b^ Leite condensado, leite condensado sabor brigadeiro, creme de chocolate com avelã e paçoca.

Nota: os valores entre parênteses são o desvio-padrão da média do grupo.

A caracterização dos AUP consistiu na descrição do valor energético, sódio, gorduras totais, gorduras saturadas e gorduras trans por 100 gramas do produto, assim como presença e número de aditivos alimentares que conferem sabor doce aos alimentos, aqui denominados “outros edulcorantes” (em alinhamento com a nomenclatura da OPAS), segundo grupo e subgrupo. Para a identificação de “outros edulcorantes”, adotou-se a definição do MPN/OPAS^7^: edulcorantes artificiais ou naturais não calóricos ou edulcorantes calóricos (polióis), não sendo considerados sucos de fruta, mel ou outros ingredientes alimentares que possam ser usados para adoçar. Os edulcorantes identificados nos rótulos dos AUP referidos foram agrupados em: edulcorantes não calóricos artificiais (aspartame, sucralose, sacarina, acessulfame de potássio, ciclamato de sódio, sacarina sódica e frutooligossacarídeos), edulcorantes não calóricos naturais (estévia) e edulcorantes calóricos, como os polióis (sorbitol, manitol, lactitol e isomalte)^7,12,13^.

Para descrição da composição nutricional do elenco de AUP referido, foram produzidas médias dos valores de energia, gorduras totais, gorduras saturadas, gorduras trans e sódio por 100 gramas ou 100 mililitros do produto, para cada grupo e subgrupo. Em relação aos “outros edulcorantes”, foram descritos, segundo grupo e subgrupo de AUP, o número de produtos com presença desses ingredientes, os números mínimo e máximo de tipos de “outros edulcorantes” por produto e, para cada grupo, os tipos de edulcorantes identificados. Para identificar se os AUP referidos apresentavam excesso de nutrientes críticos, foi utilizado o MPN/OPAS^7^, considerando-se os seguintes parâmetros: presença de “outros edulcorantes” e, em 100 gramas ou 100 mililitros dos alimentos: gorduras totais ≥ 30% da energia total, gorduras saturadas ≥ 10% da energia total, gorduras trans ≥ 1% da energia total e sódio ≥ 1 mg/kcal.

Para cada grupo de alimentos, foi calculado o percentual de AUP que ultrapassaram o limite definido pela OPAS para nutrientes críticos ou apresentaram edulcorantes na lista de ingredientes. Adicionalmente, com o objetivo de produzir uma síntese sobre a qualidade dos grupos de AUP em relação a alguns dos nutrientes críticos, os parâmetros da OPAS foram também aplicados aos valores médios de percentuais de energia provenientes de gordura total, gordura saturada, gordura trans e ao valor médio de sódio, em miligramas por calorias, observados em cada grupo. Tais resultados foram apresentados na forma de gráficos.

Por serem sujeitos a regulamentações específicas, alimentos e bebidas para fins especiais, como as fórmulas infantis e suplementos alimentares, foram excluídos da análise segundo o MPN/OPAS. Além disso, alguns temperos prontos não continham calorias, não sendo possível a avaliação do conteúdo de sódio proposta no modelo. Por esses motivos, esses produtos não foram considerados nesta análise com base no MPN/OPAS.

Segundo a legislação brasileira vigente, a presença da informação sobre alguns micronutrientes e açúcar simples não é obrigatória no rótulo dos produtos^14^. Por esse motivo, e tendo em vista a heterogeneidade da disponibilidade dessas informações nos rótulos estudados, não foi possível sua descrição nem sua análise segundo o MPN/OPAS.

Para realização dos procedimentos analíticos, foram utilizados os softwares *Statistical Package for the Social Sciences*^®^ (SPSS) versão 19 e Microsoft Excel^®^ versão 2010. O estudo principal do qual foram extraídos os dados aqui analisados foi aprovado pelo Comitê de Ética em Pesquisa com Seres Humanos da Secretaria Municipal de Saúde do Rio de Janeiro (processo nº 93/13) e contou com o apoio financeiro do CNPq (processos nº 480804/2013-3 e 420247/2016-5) e da Secretaria Municipal de Saúde do Rio de Janeiro.

## RESULTADOS

O grupo em que foram coletadas as informações sobre consumo de AUP era composto por 536 crianças. Dessas, 49,8% eram meninas e 54,3% frequentavam creches. Em relação à escolaridade materna, 20,4% possuíam ensino fundamental incompleto; 34,2%, fundamental completo; 42%, ensino médio; e 3,32%, ensino superior. No que se refere à renda familiar, 15% das famílias recebiam menos que um salário mínimo (R$ 724,00 à época); 63,9%, de um a dois; e 15,7%, três ou mais.

Para nove dos produtos referidos, os entrevistados não mencionaram marcas, ou a marca referida não foi encontrada nos mercados da cidade nem estava disponível em página do fabricante ou no serviço de atendimento ao consumidor. Foram estudados 351 AUP, sendo incluídos na análise com base no MPN/OPAS os 335 que não eram fórmulas infantis, nem suplementos alimentares, nem temperos prontos sem calorias. Os alimentos ultraprocessados de diferentes marcas e sabores referidos pelos entrevistados foram divididos em 22 grupos e 38 subgrupos. Dentre esses, destacam-se em relação ao número de tipos de alimentos referidos: bebidas açucaradas (n = 82); biscoitos (n = 60); doces e guloseimas (n = 52); iogurtes ultraprocessados e bebidas lácteas (n = 35); salgadinhos e chips (n = 14); e farinhas e espessantes (n = 14) (Tabela 1).

Quanto à composição nutricional, os grupos de AUP que apresentaram maior valor energético (em 100 gramas) foram: margarinas (597,5 kcal); salgadinhos e chips (497,4 kcal); compostos lácteos e fórmulas infantis (não reconstituídos; 489,9 kcal,); biscoitos (444,3 kcal); macarrões instantâneos (440,6 kcal); e pipocas industrializadas (418,0 kcal). Em relação às gorduras totais (em 100 gramas), os grupos com maiores teores foram: margarinas (66,3 g); salgadinhos e chips (25,6 g); compostos lácteos e fórmulas infantis (não reconstituídos; 23,3 g); pipocas industrializadas (19,8 g); macarrões instantâneos (18,2 g); e biscoitos (16,4 g). Os grupos que apresentaram teores mais elevados de gordura saturada (em 100 g) foram: margarinas (17,0 g); pipocas industrializadas (9,4 g); macarrões instantâneos (8,2 g); biscoitos (6,5 g); salgadinhos e chips (5,7 g); e temperos prontos (5,4 g). As gorduras trans foram observadas em maiores quantidades (em 100 g) nos grupos: biscoitos (0,58 g); carnes industrializadas e embutidos (0,39 g); requeijões e queijos ultraprocessados (0,33 g); e doces e guloseimas (0,29 g). Os maiores teores de sódio (em 100 g) foram observados nos grupos: temperos prontos (21.964,1 mg); macarrões instantâneos (1.816,5 mg); carnes industrializadas (773,2 mg); salgadinhos e chips (740,0 mg); e molhos industrializados e de tomate (669,8 mg), conforme apresentado na Tabela 1.

Dentre os grupos e subgrupos que apresentaram AUP com presença de “outros edulcorantes” em suas listas de ingredientes, vale destacar: bebidas açucaradas (dos 82 AUP, 26 [32%] continham edulcorantes, com destaque para refrescos em pó e bebidas saborizadas à base de extrato de soja); doces e guloseimas (11 [21%] dos 52 AUP, com destaque para gelatinas); e compostos lácteos e fórmulas infantis (3 [27%] dos 11 AUP, com destaque para os compostos lácteos). Esses resultados podem ser observados na Tabela 2. Cabe registrar que, dos 46 alimentos com presença de “outros edulcorantes”, três eram destinados a dietas especiais.

**Tabela 2:** Frequência e tipo de “outros edulcorantes” presentes nas listas de ingredientes do elenco de alimentos ultraprocessados (AUP) que continham edulcorantes consumidos por crianças de 6 a 59 meses de idade atendidas em unidades básicas de saúde. Município do Rio de Janeiro, 2014.

Alimentos ultraprocessados^a^	Nº de AUP^b^ com presença de “outros edulcorantes” (% do total de alimentos referidos)	Nº de AUP segundo número de “outros edulcorantes” presentes						Tipos de “outros edulcorantes” presentes em cada grupo e nº de AUP em que cada tipo de “outro edulcorante” estava presente
		1	2	3	4	5	6	
Bebidas açucaradas	26 (31,7)	6	7	1	12	0	0	Artificiais não calóricos (n): acessulfame de potássio (20), ciclamato de sódio (18), aspartame (14), sacarina sódica (13) e sucralose (6)
Refrescos em pó	20 (83,3)	0	7	1	12	0	0	
Bebidas saborizadas à base de extrato de soja	6 (60,0)	6	0	0	0	0	0	
Biscoitos	1 (1,7)	1	0	0	0	0	0	Naturais calóricos (*polióis*): sorbitol (1)
Biscoitos doces com recheio	1 (4,5)	1	0	0	0	0	0	
Doces e guloseimas	11 (21,2)	1	1	2	6	0	1	Artificiais não calóricos: ciclamato de sódio (10), aspartame (9), sacarina sódica (9), acessulfame de potássio (7), sucralose (1) Naturais calóricos (polióis): sorbitol (1), manitol (1)
Gelatinas	10 (100,0)	1	1	2	6	0	0	
Chicletes	1 (50,0)	0	0	0	0	0	1	
Iogurtes ultraprocessados e bebidas lácteas	1 (2,9)	0	0	0	0	0	0	Artificiais não calóricos: sucralose (1)
Leites fermentados	1 (14,3)	0	0	0	0	0	0	
Compostos lácteos e fórmulas infantis	3 (27,3)	3	0	0	0	0	0	Artificiais não calóricos: frutooligossacarídeos (3)
Fórmulas infantis	1(11,1)	1	0	0	0	0	0	
Compostos lácteos modificados	2 (100,0)	3	0	0	0	0	0	
Bolos industrializados	2 (28,6)	2	0	0	0	0	0	Naturais calóricos (*polióis*): sorbitol (2)
Bebidas à base de extrato de soja	2 (100,0)	2	0	0	0	0	0	Artificiais não calóricos: sucralose (2)

^a^ Somente grupos e subgrupos que apresentaram “outros edulcorantes” na lista de ingredientes.

^b^ Número de AUP referidos pelos entrevistados.

Em relação aos tipos de “outros edulcorantes” presentes nos grupos analisados, observou-se que as 26 bebidas açucaradas em que estes ingredientes foram identificados apresentavam de um a quatro diferentes tipos de “outros edulcorantes” em um único produto. Todos os “outros edulcorantes” identificados nas bebidas açucaradas eram artificiais não calóricos: acessulfame de potássio, ciclamato de sódio, aspartame, sacarina sódica e sucralose. No grupo de doces e guloseimas, foram identificados em um único produto seis diferentes tipos de “outros edulcorantes”. Vale destacar que as 10 gelatinas analisadas continham “outros edulcorantes”, sendo que 60% (seis) apresentavam quatro diferentes tipos. Entre os 11 alimentos do grupo doces e guloseimas que continham “outros edulcorantes”, foram identificados edulcorantes artificiais não calóricos − ciclamato de sódio, aspartame, sacarina sódica, acessulfame de potássio e sucralose – e edulcorantes naturais calóricos (polióis) − sorbitol e manitol (Tabela 2).

Dos 335 AUP analisados individualmente segundo o MPN/OPAS, 66% apresentaram “outros edulcorantes” ou excesso de pelo menos um nutriente crítico. Do total dos alimentos, 32,5% apresentaram excesso de gorduras totais; 36,4%, de gorduras saturadas; 36,7%, de sódio; 7%,de gorduras trans; e 13,4% apresentaram presença de “outros edulcorantes” em seus ingredientes. Em 13 dos 21 grupos incluídos nesta análise, todos os AUP apresentaram excesso de pelo menos um nutriente crítico. Foram eles: bebidas à base de extrato de soja; salgadinhos e chips; requeijões e queijos ultraprocessados; margarinas; compostos lácteos; carnes industrializadas e embutidos; temperos prontos; molhos industrializados e de tomate; pães; bolos industrializados; farofas industrializadas; macarrões instantâneos; e pipocas industrializadas. Os grupos de requeijões e queijos ultraprocessados, de macarrões instantâneos e de carnes industrializadas e embutidos apresentaram 100% dos alimentos com excesso de gorduras totais, de gorduras saturadas e de sódio. Os grupos de AUP que não apresentaram excesso de nenhum dos nutrientes críticos analisados foram: sucos concentrados; farinhas e espessantes; e achocolatados e saborizantes à base de morango (Tabela 3).

**Tabela 3:** Porcentagem dos alimentos ultraprocessados (AUP) que apresentaram excesso de gorduras totais, gorduras saturadas, gorduras trans e sódio em 100 gramas do produto e presença de “outros edulcorantes”a segundo grupos dos alimentos ultraprocessados consumidos por crianças de 6 a 59 meses de idade atendidas em unidades básicas de saúde. Município do Rio de Janeiro, 2014.

Grupos de alimentos ultraprocessados	N^b^	% de AUP com nutriente crítico em excesso^c^					% de AUP com pelo menos um nutriente crítico em excesso e/ou presença de “outros edulcorantes”
		Gorduras totais	Gordura saturada	Gordura trans	Sódio	Presença de “outros edulcorantes”	
Bebidas açucaradas	82	0,0	0,0	0,0	25,6	31,7	35,4
Biscoitos	60	61,7	68,3	23,3	46,7	1,7	95,0
Doces e guloseimas	52	28,8	34,6	9,6	21,2	21,2	65,4
Iogurtes ultraprocessados e bebidas lácteas	35	14,3	65,7	0,0	0,0	2,9	65,7
Salgadinhos e chips	14	92,9	14,3	0,0	78,6	0,0	100,0
Farinhas e espessantes	14	0,0	0,0	0,0	0,0	0,0	0,0
Carnes industrializadas e embutidos	11	100,0	100,0	27,3	100,0	0,0	100,0
Temperos prontos	9	44,4	44,4	0,0	100,0	0,0	100,0
Sucos concentrados	7	0,0	0,0	0,0	0,0	0,0	0,0
Requeijão e queijos ultraprocessados	7	100,0	100,0	28,6	100,0	0,0	100,0
Bolos industrializados	7	42,9	57,1	14,3	14,3	28,6	100,0
Achocolatados e saborizantes à base de morango	6	0,0	0,0	0,0	0,0	0,0	0,0
Molhos industrializados e de tomate	6	33,3	16,7	0,0	100,0	0,0	100,0
Pães	6	0,0	0,0	0,0	100,0	0,0	100,0
Margarinas	4	100,0	100,0	0,0	25,0	0,0	100,0
Cereais matinais	4	0,0	0,0	0,0	75,0	0,0	75,0
Farofas industrializadas	3	0,0	33,3	0,0	100,0	0,0	100,0
Bebidas à base de extrato de soja	2	100,0	0,0	0,0	100,0	100,0	100,0
Compostos lácteos	2	100,0	100,0	0,0	0,0	100,0	100,0
Macarrões instantâneos	2	100,0	100,0	0,0	100,0	0,0	100,0
Pipocas industrializadas	2	100,0	100,0	0,0	50,0	0,0	100,0
Total	335	32,5	36,4	7,5	36,7	13,4	66,0

^a^ De acordo com o Modelo de Perfil Nutricional da OPAS^7^.

^b^ Número de AUP referidos pelos entrevistados.

^c^ Açúcares não foram considerados nesta classificação.

A aplicação dos parâmetros da OPAS aos valores médios, em cada grupo de AUP, dos percentuais de energia provenientes de gordura total, gordura saturada e gordura trans, assim como ao valor médio de sódio em miligramas por quilocalorias, revelou que, dos 21 grupos analisados, ultrapassaram o limite estabelecido para gorduras totais 10 grupos; para gorduras saturadas, 11; para gorduras trans, 3; e para sódio, 13 (Figuras 1 e 2). Observou-se, também, que muitos grupos de AUP extrapolaram o limite estabelecido pela OPAS para gorduras totais, gorduras saturas e/ou sódio, e alguns deles (biscoitos, requeijões e queijos ultraprocessados, carnes industrializadas e embutidos) superaram os limites estabelecidos pela OPAS em todos os parâmetros.

**Figura 1:** Percentuais médios de energia proveniente de gordura total (A) e gordura saturada (B) em 100 gramas de produto por grupo de alimentos ultraprocessados consumidos por crianças de 6 a 59 meses de idade atendidas em unidades básicas de saúde comparados com faixa representando a recomendação da OPAS*. Município do Rio de Janeiro, 2014. * Modelo de Perfil Nutricional de nutrientes da OPAS^7^, que considera excesso de gorduras totais se, em determinada quantidade do alimento, a quantidade de energia (kcal) proveniente de gorduras totais (gramas de gorduras totais × 9 kcal) for igual a 30% ou mais do valor energético total (kcal) e excesso de gorduras saturadas se, em determinada quantidade do alimento, a quantidade de energia (kcal) proveniente de gorduras saturadas (gramas de gorduras saturadas × 9 kcal) for igual a 10% ou mais do valor energético total (kcal).

**Figura 2:** Percentuais médios de energia proveniente de gordura trans (A) e razão entre a quantidade de sódio (mg) e o valor energético (kcal) (B) em 100 gramas de produto por grupo de alimentos ultraprocessados consumidos por crianças de 6 a 59 meses de idade atendidas em unidades básicas de saúde com faixa representando a recomendação da OPAS*. Município do Rio de Janeiro, 2014. * Modelo de Perfil Nutricional de nutrientes da OPAS^7^, que considera excesso de gorduras trans se, em determinada quantidade do alimento, a quantidade de energia (kcal) proveniente de gorduras trans (gramas de gorduras trans × 9 kcal) for igual a 1% ou mais do valor energético total (kcal) e excesso de sódio quando a razão entre a quantidade de sódio (mg) em determinada quantidade do alimento e o valor energético (kcal) for 1:1 ou maior.

## DISCUSSÃO

Em sua maioria, os AUP referidos pelos participantes do estudo são desequilibrados nutricionalmente por conterem elevado valor energético e/ou alto teor de gorduras totais, gorduras saturadas, gorduras trans e sódio. Quando analisados segundo o MPN/OPAS, eles apresentaram, em sua maioria, excesso de pelo menos um dos nutrientes críticos e/ou presença de “outros edulcorantes” (de um a seis tipos).

Esses achados corroboram os de outros estudos que apontam que esses alimentos apresentam perfil nutricional desfavorável^15,16^. Entretanto, uma comparação mais aprofundada dos nossos achados com a literatura disponível não é tarefa simples, tendo em vista as diferenças metodológicas entre os estudos, seja em relação à classificação dos alimentos (não baseada na extensão e no propósito do seu processamento^17^) aos parâmetros para avaliação de seu perfil nutricional (critérios distintos dos da OPAS para estabelecer limites para nutrientes críticos^18^), ao grupo etário considerado (menores de dois anos^17^ ou crianças e não crianças^19^); ao enfoque da análise (por exemplo, comparação da composição nutricional de produtos dirigidos ao público infantil, com e sem alegação de saúde^9^), ou à base empírica (por exemplo, produtos disponíveis no mercado^8^).

Em que pesem essas diferenças, outros estudos apontam resultados convergentes com os encontrados aqui. No Canadá, em 2005, uma análise do perfil nutricional dos produtos direcionados para crianças em um supermercado encontrou que, dos 327 produtos analisados, 89% poderiam ser classificados como de má qualidade nutricional (quantidades elevadas de açúcar, gordura e/ou sódio)^18^. Um resultado na mesma direção foi observado em um estudo realizado na Austrália em 2009: dos 157 produtos comercializados para crianças avaliados, 75,2% apresentavam alta concentração de gordura ou açúcar^20^.

Estudos que compararam AUP direcionados para o grupo infantil e não infantil revelaram que os primeiros não apresentaram composição nutricional mais favorável. Uma pesquisa realizada em 2013 em Florianópolis comparou os produtos direcionados para esses dois grupos e identificou que achocolatados, biscoitos doces com e sem recheio, guloseimas, refrigerantes e salgadinhos dirigidos para crianças apresentaram menor quantidade de gorduras totais, menor quantidade de fibras e maior quantidade de sódio do que aqueles dirigidos para outros grupos etários^21^. Já um estudo realizado no Reino Unido entre 2010 e 2011 apontou que um número significativo de produtos comercializados para as crianças apresentava maiores teores de gordura, açúcar e sal do que aqueles comercializados para a população em geral^19^.

No documento da OPAS que propõe os critérios para análise de nutrientes críticos, comparou-se o perfil nutricional de alguns AUP direcionados a todos os públicos entre diversos países^7^. No caso do Brasil, os grupos de AUP em que 100% dos produtos analisados apresentaram teores excessivos de um ou mais nutrientes críticos foram: cereais matinais; pães embalados; iogurte com adição de açúcar; leite com adição de açúcar; sorvetes; presuntos; caramelos e chocolates; e bebidas adoçadas com açúcar^7^. Os dados foram semelhantes aos nossos achados quanto ao teor excessivo de pelo menos um nutriente crítico em todos os pães embalados, presuntos, leites com adição de açúcar e caramelos e chocolates analisados.

Pelo fato de o MPN/OPAS ter sido publicado recentemente, são ainda poucos os estudos que aplicaram os parâmetros nele propostos. Em uma pesquisa realizada em 2014 no Uruguai que o fez, observou-se que todos os AUP direcionados a crianças disponíveis em supermercados em Montevidéu apresentavam teores excessivos de pelo menos um nutriente crítico^8^. Comparados com os resultados de nossa pesquisa, o grupo dos biscoitos e bolos e o grupo dos produtos lácteos apresentaram perfil nutricional pior naquele país^8^. Na Argentina, um estudo realizado em 2014 que avaliou o número de anúncios de alimentos processados e ultraprocessados direcionados a crianças em canais abertos de televisão encontrou que, de 2.959 anúncios, 93,1% anunciavam produtos com teores excessivos de energia e/ou um ou mais nutrientes críticos^22^. Um estudo realizado no Canadá em 2017 identificou que, de 365 produtos processados e ultraprocessados voltados para crianças disponíveis em um supermercado, 86,5% apresentavam excesso de açúcares livres; 36,2%, excesso de sódio; 32,5%, excesso de gordura saturada; e 28,7%, excesso de gordura total^23^.

Os resultados sobre a composição nutricional dos AUP são preocupantes por sua relação com desfechos desfavoráveis para a saúde^6^. Além disso, as vendas e o consumo desses AUP estão aumentando em todo o mundo nos últimos anos, sendo o consumo recorrente entre crianças^3,4,5^.

Em relação aos edulcorantes, o consumo habitual de produtos com sabor doce por crianças tem sido associado com uma menor qualidade da dieta, definindo os padrões de consumo ao longo da vida^24^. Adicionalmente, alguns estudos sugerem que a exposição precoce a edulcorantes (adoçantes calóricos e não calóricos) pode afetar adversamente a composição corporal, a saúde cardiometabólica e a microbiota intestinal^24,25^.

O presente estudo apresenta algumas limitações. Alguns dos entrevistados não referiram as marcas dos produtos consumidos ou referiram marcas que não foram encontradas nos mercados da cidade nem estavam disponíveis na página do fabricante ou no serviço de atendimento ao consumidor. Esses fatores podem ter levado à subestimação do elenco de produtos analisados e, portanto, a erros (para mais ou para menos) na composição nutricional dos grupos de AUP analisados.

Outro aspecto que merece ser comentado é o fato de características da legislação de rotulagem no Brasil terem limitado as análises referentes a açúcares. O fato de essa informação não ser obrigatória^14^ faz com que ela não conste nos rótulos de muitos produtos. Isso impediu a análise do parâmetro “excesso de açúcares livres”, previsto no MPN/OPAS. A consequência disso foi a não caracterização desse parâmetro para grupos de AUP que foram recorrentemente referidos pelo grupo estudado e nos quais o açúcar é um dos principais ingredientes: cereais matinais; bolos industrializados; iogurtes ultraprocessados e bebidas lácteas; doces e guloseimas; bebidas açucaradas; farinhas e espessantes; e achocolatados e saborizantes à base de morango. Além disso, na legislação brasileira vigente, a rotulagem nutricional obrigatória sobre gordura trans se refere à porção do produto (e não a 100 g) e aqueles com teores inferiores ou iguais a 0,2 g por porção não necessitam relatar a presença desse nutriente^14^. Com isso, a presença e o teor de gordura trans no conjunto de produtos referidos pelos participantes podem ter sido subestimados. Ou seja, o perfil nutricional dos AUP referidos pelo grupo estudo pode ser ainda menos adequado do que o descrito aqui.

Cabe ainda comentar o fato de os dados analisados serem oriundos de somente um R-24h. Esse procedimento é adequado, uma vez que o objetivo do estudo foi o de analisar a composição nutricional do elenco de alimentos referidos pelos participantes e não o de quantificar, em nível individual, o consumo de macro e micronutrientes e de associá-lo a outros eventos de interesse, propósito que poderia justificar a realização de pelo menos dois R-24h^26^. A realização das entrevistas entre terça e sexta-feira forneceu um panorama conservador da alimentação das crianças, uma vez que a presença de AUP em fins de semana pode ser ainda maior.

Entre os pontos fortes do estudo, merece destaque o fato de os produtos analisados serem aqueles referidos por uma amostra representativa de crianças menores de cinco anos usuárias do SUS em uma grande cidade brasileira. Essa escolha oferece duas vantagens em relação à abordagem mais recorrente na literatura, que é a análise dos produtos disponíveis em mercado(s) local(is)^8,18,19,21^. A primeira delas é subsidiar políticas públicas dirigidas a esse grupo a serem implementadas na rede pública de atenção básica, pois permite a caracterização dos AUP efetivamente consumidos por ele. A segunda vantagem é oferecer um panorama de quais são os AUP de fato consumidos por crianças, independentemente de serem dirigidos a esse público, o que pode subsidiar medidas regulatórias mais abrangentes, como a regulação do preço, da rotulagem e da publicidade desses alimentos.

Outro aspecto a ser valorizado é a análise realizada com base no MPN/OPAS^7^, ainda incipiente na literatura^8,22,23^ e, até onde se tem notícia, pioneira no Brasil. Ainda sobre essa análise, também merece ser comentado o exercício de aplicação dos parâmetros da OPAS à média dos percentuais de nutrientes críticos examinados. Isso permitiu produzir uma interpretação complementar à recomendada pela OPAS, que oferece uma visão geral dos grupos de alimentos em relação a esses parâmetros.

Conclui-se que o presente estudo revelou que os AUP consumidos por crianças usuárias do SUS na cidade do Rio de Janeiro apresentam perfil nutricional inadequado e, em sua maioria, teores excessivos de pelo menos um nutriente crítico para problemas de saúde pública. Além disso, uma parcela deles contém pelo menos um “outro edulcorante”. Essas evidências sugerem que, além de ações educativas que estimulem a preservação da cultura alimentar brasileira, para frear o aumento do consumo dos AUP, é urgente a difusão do Guia Alimentar da População Brasileira, que recomenda evitar o consumo desses alimentos, bem como a implementação de medidas regulatórias que: (a) restrinjam práticas mercadológicas que incentivem seu consumo; (b) tributem os AUP, aumentando seu preço final; (c) melhorem a rotulagem dos alimentos, com informações nutricionais mais claras e rotulagem frontal de advertência em relação a nutrientes críticos e edulcorantes, à luz do que vem sendo discutido no âmbito da ANVISA^27^; e (d) restrinjam a oferta de AUP e ampliem a oferta de alimentos *in natura* ou minimamente processados no espaço escolar.

São necessários estudos futuros que ampliem as evidências sobre os AUP, seus ingredientes, seu consumo em diferentes grupos populacionais, sua relação com desfechos de saúde e, também, que os analisem sob a perspectiva do MPN/OPAS.
